# Transcription Factor CREB3L1 Regulates Endoplasmic Reticulum Stress Response Genes in the Osmotically Challenged Rat Hypothalamus

**DOI:** 10.1371/journal.pone.0124956

**Published:** 2015-04-27

**Authors:** Mingkwan Greenwood, Michael Paul Greenwood, Julian F. R. Paton, David Murphy

**Affiliations:** 1 School of Clinical Sciences, University of Bristol, Bristol, England; 2 School of Physiology and Pharmacology, University of Bristol, Bristol, England; 3 Department of Physiology, University of Malaya, Kuala Lumpur, Malaysia; National Institutes of Health, UNITED STATES

## Abstract

Arginine vasopressin (AVP) is synthesised in magnocellular neurons (MCNs) of supraoptic nucleus (SON) and paraventricular nucleus (PVN) of the hypothalamus. In response to the hyperosmotic stressors of dehydration (complete fluid deprivation, DH) or salt loading (drinking 2% salt solution, SL), AVP synthesis increases in MCNs, which over-burdens the protein folding machinery in the endoplasmic reticulum (ER). ER stress and the unfolded protein response (UPR) are signaling pathways that improve ER function in response to the accumulation of misfold/unfold protein. We asked whether an ER stress response was activated in the SON and PVN of DH and SL rats. We observed increased mRNA expression for the immunoglobulin heavy chain binding protein *(BiP)*, activating transcription factor 4 *(Atf4)*, C/EBP-homologous protein *(Chop)*, and cAMP responsive element binding protein 3 like 1 (*Creb3l1)* in both SON and PVN of DH and SL rats. Although we found no changes in the splicing pattern of X box-binding protein 1 (*Xbp1*), an increase in the level of the unspliced form of *Xbp1* (*Xbp1*U) was observed in DH and SL rats. CREB3L1, a novel ER stress inducer, has been shown to be activated by ER stress to regulate the expression of target genes. We have previously shown that CREB3L1 is a transcriptional regulator of the AVP gene; however, a role for CREB3L1 in the response to ER stress has yet to be investigated in MCNs. Here, we used lentiviral vectors to introduce a dominant negative form of CREB3L1 (CREB3L1DN) in the rat SON. Expression of CREB3L1DN in the SON decreased *Chop* and *Xbp1*U mRNA levels, but not *BiP *and* Atf4 *transcript expression. CREB3L1 is thus implicated as a transcriptional mediator of the ER stress response in the osmotically stimulated SON.

## Introduction

Water homeostasis is regulated by the neuropeptide hormone arginine vasopressin (AVP), which is synthesised by magnocellular neurons (MCNs) of the supraoptic nucleus (SON) and the paraventricular nucleus (PVN) of the hypothalamus. The mRNA encoding AVP is translated in MCN cell bodies into a preprohormone that is delivered into the endoplasmic reticulum (ER) with concomitant signal peptide removal. The resulting prohormone is folded, and then delivered to the Golgi apparatus where it is targeted to large dense core vesicles, which are then transported some considerable distance to storage in the posterior pituitary, during which time the precursor is cleaved and post-translationally modified into the mature hormone [1]. A rise in plasma osmolality is sensed by specialised osmosensitive circumventricular organs that project to MCNs, providing direct excitatory inputs that shape neuronal firing, leading to hormone secretion from the posterior pituitary [2, 3]. Once released, AVP travels through blood stream to the kidney where it provokes water re-absorption [4]. An increase in plasma osmolality, as elicited by complete fluid deprivation (dehydration, DH) or the obligatory ingestion of 2% (w/v) NaCl (salt loading, SL), results in increased AVP biosynthesis in MCNs of the SON and PVN, as evidenced by increased AVP gene transcription and an increase in the abundance of AVP mRNAs [5, 6].

The ER is a tubular organelle which is crucially responsible for the proper folding of proteins that are destined to be secreted from cells. Dysfunction of ER, or a large demand for protein synthesis under stressful conditions, can lead to accumulation of unfolded or misfolded proteins in the ER lumen, which induces ER stress. To overcome ER stress, cells activate a signaling pathway, known as the unfolded protein response (UPR), which helps to recover ER function through the activation of the expression of chaperone proteins such as the immunoglobulin heavy chain binding protein (BIP) to facilitate protein folding, inhibit general protein translation to reduce workload in the ER, and increase degradation of misfolded proteins [7].

The activation of the UPR is modulated through three ER stress sensors: inositol-requiring protein 1α (IRE1α), protein kinase RNA-like endoplasmic reticulum kinase (PERK), and activating transcription factor 6 (ATF6) [8]. Immediate activation of PERK inhibits general protein translation and allows translation of activating transcription factor 4 (ATF4), a UPR-downstream gene which regulates transcription of pro-apoptotic transcription factor C/EBP-homologous protein (*Chop*) [9]. Activation of IRE1α results in the selective splicing of the mRNA encoding the transcription factor X box-binding protein 1 (*Xbp1*). Excision of a 26-nucleotide-long intron creates a spliced form of *Xbp1* mRNA (*Xbp1*S) [10–12], resulting in expression of an active and stable transcription factor that regulates transcription of target genes involved in protein folding and ER-associated degradation (ERAD) [13, 14]. ER stress also induces translocation of ATF6, a basic leucine zipper (bZIP) transcription factor, to the Golgi apparatus where it is processed by regulated intramembrane proteolysis (RIP) [15]. The cytosolic fragment of ATF6 is then transported into the nucleus to control the transcription of genes in ERAD components and XBP1 [11, 16].

Recently, a novel ER stress transducer CREB3L1 (cAMP responsive element binding protein 3 like 1; also known as OASIS), that share a region of high sequence similarity to ATF6, has been identified [17]. This protein consists of a transmembrane domain that allows it to associate with the ER, a bZIP domain and a transcription activation domain [18]. Moreover it contains a consensus sequences for RIP cleavage as found in ATF6, suggesting that both transcription factors are activated by this process. CREB3L1 was previously shown to be activated by ER stress in various physiological models, and functions in the regulation of target genes that are important to those physiological conditions [19–21]. Recently, we showed that CREB3L1 is able to increases AVP transcription in AVP MCNs of the SON and PVN [22]. However, the function of CREB3L1 related to ER stress in the hypothalamus in response of hypertonic stress has not been investigated.

The expression of *BiP*, a component of the UPR pathway, has been shown to increase in expression in the SON and PVN following osmotic stimulation [23], however detailed investigations on ER stress in these neurons are lacking. Transcriptome analysis using microarray has revealed an increase in expression of a number of ER stress-related genes in the SON of 3 days DH rats [24]. This included well known ER stress related genes, such as *Atf4* and *Chop*, and also the novel ER stress transducer *Creb3l1*. In order to meet physiological demand, hyperosmotic events provoke large increases in AVP prohormone synthesis. We hypothesized that the presence of considerable quantities of AVP prohormone in the ER of MCNs might increase the demands placed on the protein folding machinery in the ER of these cells. This may activate physiological ER stress and UPR pathways in order to improve protein folding capacity, as previously reported in other physiological models [19–21]. Thus, in this study, we characterized the ER stress response in MCNs of rat hypothalamus in response to hyperosmotic cues. Further, we used lentiviral over-expression of a dominant negative CREB3L1 mutant (CREB3L1DN) in the SON to demonstrate that CREB3L1 has a role in the regulation of ER stress-related genes under hyperosmotic conditions.

## Materials and Methods

### Animals

Male Sprague-Dawley rats weighing 250–300 g were used in this study. The rats were housed at a constant temperature of 22°C and a relative humidity of 50–60% (v/v) under a 14:10h light/dark cycle. The rats were given free access to food and tap water for at least 1 week prior to experimentation. To induce hyperosmotic stress water was removed for 1 or 3 days (DH) or replaced by 2% (w/v) NaCl in drinking water for 1 or 7 days (SL). All rats were humanely killed by striking of the cranium (stunning) and brains were removed and frozen on dry ice before being stored at -80°C. All experiments were performed under a Home Office UK licence held under, and in strict accordance with, the provision of the UK Animals (Scientific Procedures) Act (1986); they had also been approved by the University of Bristol Ethical Review Committee.

### RNA extraction and cDNA synthesis

Rats were stunned and decapitated and brains were removed and immediately frozen in powdered dry ice. The SON and PVN samples were collected using a 1 mm micropunch (Fine Scientific Tools) from 60 μm coronal sections in a cryostat. Sections were mounted on glass slides and stained with 0.1% (w/v) toludine blue, then visualised on a light microscope until the desired brain nuclei was visible. Samples were punched from frozen brain slices and dispensed into 1.5 ml tubes kept on dry ice within the cryostat. Total RNA was extracted from punch samples by combining Qiazol Reagent with Qiagens RNeasy kit protocols (Qiagen). The punch samples were removed from dry ice and rapidly resuspended, by vortexing, in 1 ml Qiazol reagent. Following Qiazol phase separation with chloroform, 350 μl of the upper aqueous phase was removed, mixed with 350 μl 70% ethanol and applied to RNeasy columns. The remaining steps were performed as recommended by the manufacturer. For cDNA synthesis, 100–200 ng of total RNA was reverse transcribed using the Quantitect reverse transcription kit (Qiagen).

### Real-time quantitative PCR analysis

**Fig 1:**
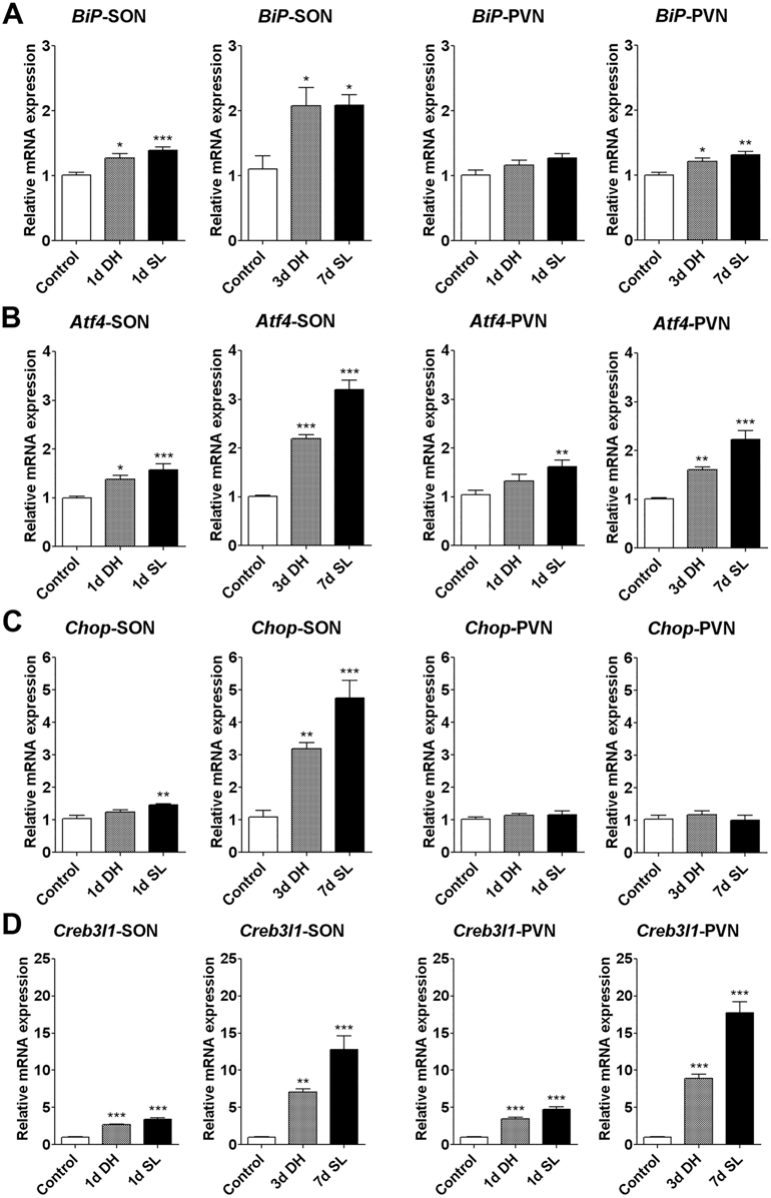
UPR-target genes increase in expression in response to hyperosmotic stress. Relative mRNA expression of (A) *BiP*, (B) *Atf4*, (C) *Chop*, and (D) *Creb3l1* in the SON and PVN of 1 day and 3 days DH and 1 day and 7 days SL rats was examined by qPCR (n = 5–8). Error bar, +SEM, * *p*<0.05, ** *p*<0.01, *** *p*<0.001. d, day; DH, dehydration; SL, salt loading; SON, supraoptic nucleus; PVN, paraventricular nucleus.

Primers for *BiP* (5´-GACCACCTATTCCTGCGTCGGT-3´ and 5´- CGCCAATCAGACGCTCCCCT -3´), *Creb3l1* (used in Fig 1) (5´-GAGACCTGGCCAGAGGATAC-3´ and 5´-GTCAGTGAGCAAGAGAACGC-3´), *Creb3l1* (used in viral transduction experiment) (5´-GCCAACAGGACCCTGCTCCA-3´ and 5´-AGTGCCAGTCTGTGTGGCCG-3´), *Xbp1*S (5´-GCTTGTGATTGAGAACCAGG-3´ and 5´-GGCCTGCACCTGCTGCGGACTC-3´) [25], *Xbp1*U (5´-GCCCTGGTTACTGAAGAGGT-3´ and 5´-CACGTAGTCTGAGTGCTGC-3´), *Rpl19* (5'-GCGTCTGCAGCCATGAGTA-3' and 5'-TGGCATTGGCGATTTCGTTG-3'), *Gapdh* (5’-ATGATTCTACCCACGGCAAG-3’ and 5’-CTGGAAGATGGTGATGGGTT-3’), were synthesised by European MWG Operon, while rat *Atf4* and *Chop (Ddit3)* primers were purchased from Quantitect Primer Assays (Qiagen). The optimisation and validation of primers was performed using standard ABI protocols. The cDNA from RT reaction was used as a template for subsequent PCRs, which were carried out in duplicate using an ABI 7500 Sequence Detection System (ABI, Warrington, UK). For relative quantification of gene expression the 2^-ΔΔCT^ method was employed [26]. The internal control genes used for these analyses were the housekeeping genes *Rpl19* and *Gapdh*.

### Immunofluorescence

Rats were deeply anesthetised with sodium pentobarbitone (100 mg/kg, intraperitoneal) and transcardially perfused with 0.1 M phosphate buffered saline pH 7.4 (PBS) followed by 4% (w/v) paraformaldehyde (PFA) in PBS. The brains were removed and post-fixed overnight in 4% (w/v) PFA followed by 30% (w/v) sucrose prepared in PBS to cryoprotect the tissue prior to freezing over liquid nitrogen. Coronal sections (40 μm) of the forebrain were cut on a cryostat and washed in PBS three times. Sections then were blocked in 5% fetal bovine serum prepared in 0.25% (v/v) TritonX/PBS (PBST) for 30 min and then incubated with appropriate primary antibodies at 4°C for 48 hours. The sections were washed three times in PBS for 5 min and incubated with 1:500 dilution of appropriate biotinylated secondary antibody in PBST for 1 hour at room temperature. The sections were washed three times for 5 min with PBS and incubated for 1 hour with secondary antibodies conjugated with fluorophore (Alexa Fluor 488 streptavidin-conjugated and Alexa Fluor 594 donkey anti-mouse or rabbit IgG (Invitrogen)). After three washes with PBS, sections were mounted onto glass slides with 0.5% gelatin, and sealed with VectorShields hard mounting media (Vector Laboratories Ltd.). The following antibodies were used for immunostaining: rabbit polyclonal anti-neurophysin II (AVP; 1:1,000; Sigma N0774), mouse monoclonal anti-CHOP antibody (1:200; Abcam ab11419) and goat anti-N-terminal CREB3L1 antibody (1:200; R&D systems).

### Construction of Lentiviruses

Lentivirus production and viral injections into rat SON were performed as previously described [22]. Plasmid construct expressing CREB3L1DN mutant of mouse CREB3L1 cloned into pcDNA3 was generously provided by Kazunori Imaizumi (University of Miyazaki). A cDNA clone encoding CREB3L1DN was cloned into lentiviral transfer vector pRRL.SIN.CPPT.CMV.IRES-GFP.WPRE. A lentiviral vector expressing GFP (pRRL.SIN.CPPT.CMV.GFP.WPRE) was used as a control (Addgene). The transfer vectors and three separate packaging plasmids (pMDLg/pRRE, pRSV-Rev, PMD2.G (Addgene)) were propagated in Stbl3 competent cells (Invitrogen) and purified using PureLink HiPure Plasmid Filter Maxiprep kit (Invitrogen). Viruses were generated by transient transfection of the transfer vector together with packaging plasmids into HEK293T/17 cells (human embryonic kidney cell line, ATCC CRL-11268) by calcium phosphate method. Culture supernatant containing lentivirus was collected at 48 and 72 hours after transfection, cell debris was removed by centrifugation, and the supernatant was filtered through 0.45 μm filter (Corning, 430770). The viruses were concentrated by centrifugation at 6000×g for 16 hours (400 ml), followed by ultracentrifugation of the resuspended pellet (10 ml PBS) for 1.5 hours at 50,000×g. The viral pellet was resuspended in 150 μl of pre-warmed PBS, aliquoted and stored at -80°C. Viral titers were determined by counting GFP positive cells at day 3 following infection of serially diluted virus in HEK293T/17 cells.

### Lentiviral vector gene transfer into SON

For bilateral SON injection, rats were anesthetised by intraperitoneal administration of Domitor/Ketamine and placed in a stereotaxic frame in the flat skull position. A 2 cm rostral-caudal incision was made to expose the surface of the skull. Two 1 mm holes were drilled at co-ordinates 1.3 mm posterior to Bregma and 1.8 mm lateral to midline. A 5 μl pulled glass pipette was positioned -8.8 mm ventral to the surface of the brain and 1 μl of lentiviral vector was delivered into nuclei over 10 min. The glass pipette was fixed in position for a further 5 minutes to minimise back tracking of the virus. Following injections, the incision was closed and Antisedan was administered intramuscularly. After surgery, animals were individually housed in standard laboratory cages for two weeks. Rats were subjected to 3 days DH, and then killed by stunning and decapitation. Brains were immediately frozen on dry ice and stored at -80°C.

### Statistical analysis

All data are expressed as the mean +SEM. Statistical differences between experimental groups were evaluated using independent sample unpaired Student’s *t*-tests or One-way ANOVA (for comparison of more than two groups). P<0.05 was considered significant.

## Results

### Expression of ER stress-responsive genes in SON and PVN of DH and SL rats

One of the methods used to monitor ER stress/UPR activation is to examine the expression of UPR-target genes [27, 28]. To see whether ER stress in activated in response to hyperosmotic stress in rat SON and PVN, the mRNA expression level of *BiP*, *Atf4*, and *Chop* was examined by qPCR (Fig 1A–1C). Expression of *BiP* and *Atf4* significantly increased in SON of 1 day DH (*BiP* 1.272+0.065, *p*<0.05; *Atf4* 1.383+0.071, *p*<0.05) and 1 day SL (*BiP* 1.389+0.051, *p*<0.001; *Atf4* 1.575+0.122, *p*<0.001) compared to control rats (*BiP* 1.006+0.049; *Atf4* 1.001+0.023), whilst expression of *Chop* was significantly increased in 1 day SL, but not DH (control 1.035+0.11; 1d DH 1.246+0.067; 1d SL 1.452+0.044, *p*<0.01). The magnitude of increase was higher for the longer time points of 3 days DH (*BiP* 2.077+0.28, *p*<0.05; *Atf4* 2.19+0.083, *p*<0.001; *Chop* 3.189+0.192, *p*<0.01) and 7 days SL (*BiP* 2.086+0.16, *p*<0.05; *Atf4* 3.193+0.195, *p*<0.001; *Chop* 4.742+0.554, *p*<0.001; Control: *BiP* 1.102+0.206, *Atf4* 1.002+0.029, *Chop* 1.095+0.189).

The level of increase of *BiP* and *Atf4* mRNA in PVN was smaller than that observed in SON. No significant difference in *BiP* mRNA in PVN of both 1d DH and 1d SL rats (control 1.013+0.072; 1d DH 1.162+0.073; 1d SL 1.271+0.073) was observed, whilst *Atf4* only increased in expression following 1 day SL, but not DH (control 1.039+0.095; 1d DH 1.325+0.135; 1d SL 1.619+0.137, *p*<0.01). A significant increase of *BiP* and *Atf4* mRNAs was observed at the later time points of 3 day DH and 7 day SL (*BiP*: control 1.004+0.04; 3d DH 1.209+0.052, *p*<0.05; 7d SL 1.312+0.056, *p*<0.01; *Atf4*: control 1.002+0.03; 3d DH 1.610+0.052, *p*<0.01; 7d SL 2.227+0.184, *p*<0.001). *Chop* expression was unaltered in PVN after 1 day (control 1.019+0.068; 1d DH 1.131+0.053; 1d SL 1.159+0.12) and at later time points (control 1.039+0.109; 3d DH 1.174+0.113; 7d SL 0.999+0.156) (Fig 1A–1C).

As previously reported [22], and shown here in a new set of experimental data, a significant increase of *Creb3l1* mRNA was seen in both SON and PVN at 1 day DH and SL (SON: control 1.013+0.074; 1d DH 2.659+0.138, *p*<0.001; 1d SL 3.377+0.268, *p*<0.001; PVN: control 1.021+0.083; 1d DH 3.482+0.171, *p*<0.001; 1d SL 4.751+0.367, *p*<0.001). A greater magnitude increase was observed at the longer time points (SON: control 1.015+0.077; 3d DH 7.102+0.402, *p*<0.01; 7d SL 12.82+1.812, *p*<0.001; PVN: control 1.013+0.072; 3d DH 8.885+0.588, *p*<0.001; 7d SL 17.76+1.437, *p*<0.001).

### Increased expression of the unspliced but not the spliced form of XBP1 in response of hyperosmotic stress

**Fig 2:**
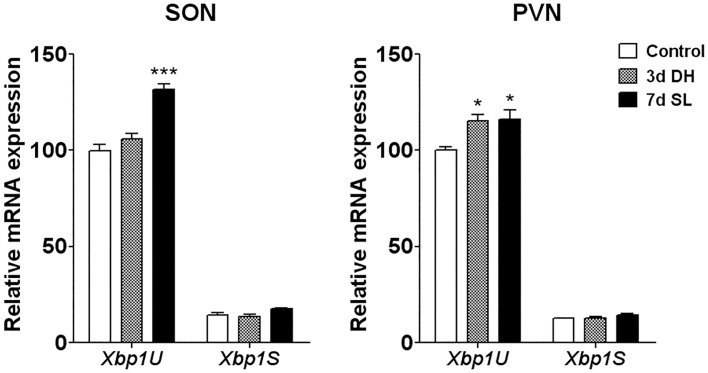
*Xbp1* splicing in the SON and PVN of 3 days DH and 7 days SL rats. Splicing of *Xbp1* mRNA was investigated in the SON and PVN of 3 days DH and 7 days SL rats. Relative mRNA expression of *Xbp1*U and *Xbp1*S mRNA in the SON and PVN of 3 days DH and 7 days SL rats was examined by qPCR (n = 6). The data is presented as percentage of relative mRNA expression where control *Xbp1*U is 100%. Error bar, +SEM, * *p*<0.05, *** *p*<0.001. d, day; DH, dehydration; SL, salt loading; *Xbp1*U, unspliced form of *Xbp1* mRNA; *Xbp1*S, spliced form of *Xbp1* mRNA; SON, supraoptic nucleus; PVN, paraventricular nucleus.

To investigate the activation of ER stress pathways in MCNs of SON and PVN in response to hyperosmotic stress, we examined the splicing patterns of *Xbp1* in 3 day DH and 7 day SL compared to control rats using primers specific to spliced (*Xbp1*S) or unspliced (*Xbp1*U) forms of *Xbp1* mRNA. Quantitative analysis of *Xbp1*S and *Xbp1*U was performed by qPCR (Fig 2). The percentage of relative mRNA expression level of *Xbp1*S and *Xbp1*U was calculated relative to the level of *Xbp1*U in the control samples. A significant increase of *Xbp1*U mRNA was observed in the SON of 7 days SL (131.5% +3.129, *p*<0.001), but not 3 days DH (105.9% +3.047), while *Xbp1*U mRNA increased in PVN of 3 days DH (115.2% +3.251, *p*<0.05) and 7 days SL (116.3% +4.885, *p*<0.05) rats compared to control (SON: 100.0% +3.251; PVN: 100.0% +1.910). A low level of *Xbp1*S mRNA was observed in both SON and PVN of control rats by qPCR (SON 14.50% +1.160; PVN 12.58% +0.264). No significant change in the abundance of *Xbp1*S was observed in the SON and PVN in response to 3 days DH (SON 13.75% +0.958; PVN 12.91% +0.565) nor 7 days SL (SON 17.58% +0.629; PVN 14.45% +0.717) (Fig 2).

### Effect of CREB3L1DN over-expression on ER-stress responsive genes in SON of dehydrated rats

**Fig 3:**
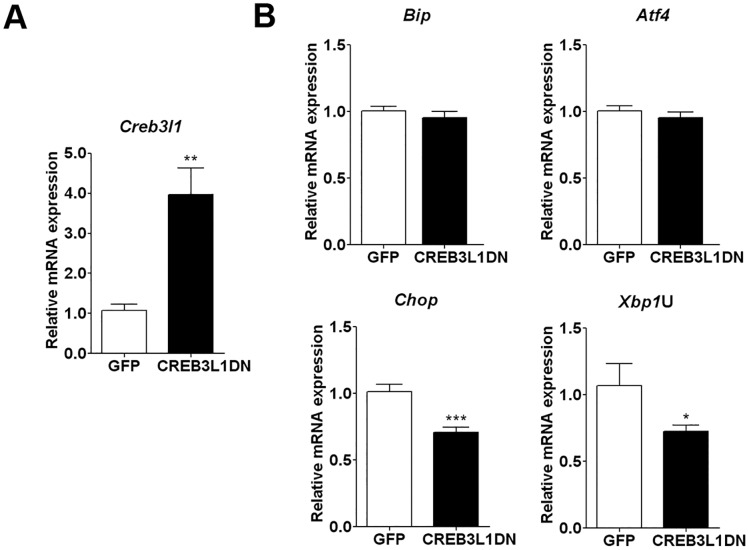
Effect of CREB3L1DN expression on UPR-target genes in rat SON. Rats were bilaterally injected into SON with lentiviral vectors expressing either CREB3L1DN or GFP. Two weeks after injection the rats were subjected to 3 days DH. The relative mRNA expression of (A) *Creb3l1*, (B) *BiP*, *Atf4*, *Chop*, and *Xbp1*U was examined by qPCR (GFP n = 7, CREB3L1DN n = 10). Error bar, +SEM. * p<0.05, ** *p*<0.01, *** *p*<0.001. d, day(s); DH, dehydration; SL, salt loading; SON, supraoptic nucleus; PVN, paraventricular nucleus; AVP, arginine vasopressin; CREB3L1DN, a dominant negative form of CREB3L1.

**Fig 4:**
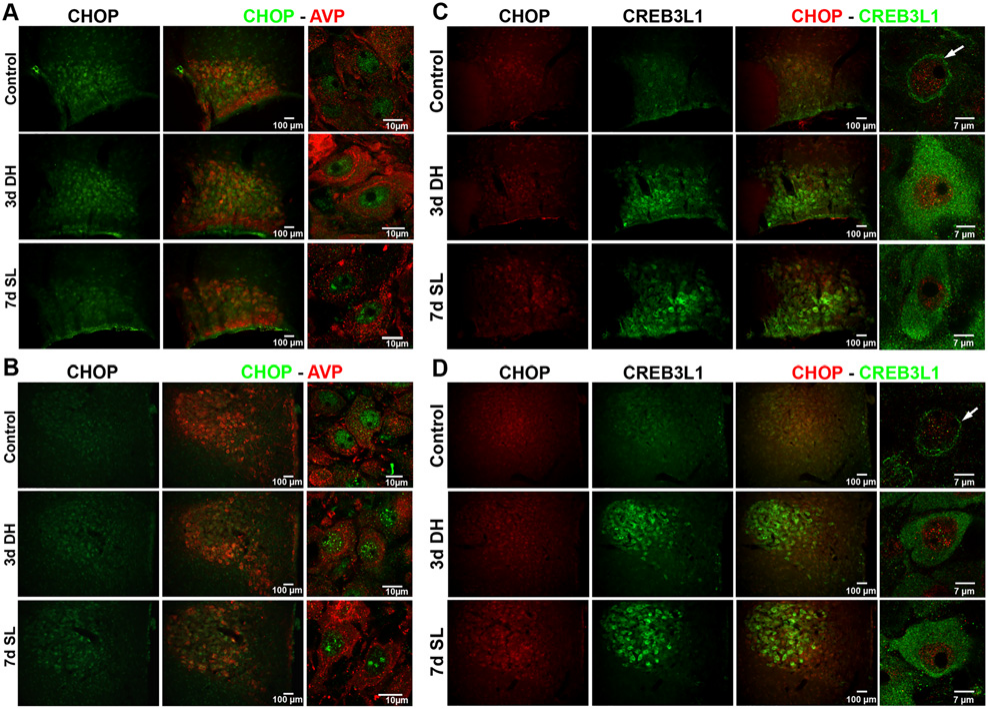
Localisation of CHOP in the SON and PVN. (A-B) Immunostaining of CHOP (green) and AVP (red) in the (A) SON and (B) PVN of 3 days DH and 7 days SL rats shows expression of CHOP in the nuclei of AVP MCNs. (C-D) Immunostaining of CHOP (red) and CREB3L1 (green) in the (C) SON and (D) PVN of 3 days DH and 7 days SL rats shows co-existence of CHOP and CREB3L1 in the same MCNs. High magnification confocal microscope images shows a change in localisation of CREB3L1 from perinuclear staining in control to cytoplasmic and nuclear staining in both 3 days DH and 7 days SL. Arrows indicate perinuclear staining of CREB3L1. d, day; DH, dehydration; SL, salt loading; AVP, arginine vasopressin.

To investigate the role of CREB3L1 in the elaboration of ER stress in the osmotically stimulated SON, we used lentiviral vector-mediated gene delivery to over-express CREB3L1DN in the rat SON. Two weeks after bilateral injection of the virus into the SON, rats were subjected to water deprivation for 3 days. qPCR analysis of *Creb3l1* showed successful expression of CREB3L1DN in the SON (Fig 3A; control virus expressing GFP 1.074+0.152; CREB3L1DN 3.971+0.655, *p* = 0.0025). The expression of *BiP*, *Atf4*, *Chop* and *Xbp1*U mRNA were then quantified following knockdown of CREB3L1 activity. No change in the expression of *BiP* or *Atf4* was observed, whilst *Chop* and *Xbp1*U mRNA expression was significantly decreased in CREB3L1DN transduced SON compared to control GFP animals (Fig 3B; *BiP* GFP 1.004+0.036, CREB3L1DN 0.955+0.045, *p* = 0.441; *Atf4* GFP 1.005+0.039, CREB3L1DN 0.955+0.040, *p* = 0.41; *Chop* GFP 1.011+0.057, CREB3L1DN 0.709+ 0.037, *p* = 0.0003; *Xbp1*U GFP 1.069+ 0.165, CREB3L1DN 0.724+ 0.048, *p* = 0.0337). Immunostaining of CHOP protein with AVP and CREB3L1 was investigated to support a relationship between CREB3L1 and CHOP. The result showed CHOP protein staining in the nuclei of AVP MCNs in both SON and PVN of control, 3 days DH and 7 days SL rats, although a change in protein expression level was not clear. The localisation of CHOP protein in AVP MCNs, supports a role for this gene in the MCNs (Fig 4A and 4B). Double-immunolabelling of CREB3L1 and CHOP also showed co-existence of these genes in the MCNs in both SON and PVN (Fig 4C and 4D). A change in subcellular localization of CREB3L1 in the MCNs in response of hyperosmotic stress was observed here similar to what we previously reported [22]. CREB3L1 staining was seen in the perinuclear area of MCNs in control rats, while staining was observed in the cytoplasm and nucleus of MCNs of DH and SL rats.

## Discussion

Using Affymetrix oligonucleotide microarrays, we have compiled comprehensive lists of mRNAs in the SON and PVN that change in abundance in response to hyperosmotic stress in the rat [24]. Examination of these datasets revealed increased expression of a number of important regulatory components of the UPR pathway, which prompted us to validate these data by qPCR. Thus, we report here increased *BiP*, *Atf4*, *Chop*, *Xbp1*U and *Creb3l1* mRNA expression in SON and/or PVN of DH and/or SL rats compared to control rats, suggesting that ER stress is activated in response to hyperosmotic stress. Most ER stress genes examined in this study showed smaller changes in mRNA expression in the PVN in response to DH and SL compared to SON. We suggest that this may be a consequence of the SON being a homogenous population of MCNs, dedicated to the elaboration of large quantities of AVP, whilst the PVN is heterogeneous, comprising both MCNs making AVP, and parvocellular neurons with different functions [29].

Here we propose that the increased demand for protein synthesis in AVP MCNs of the hypothalamus during hypertonic stress leads to disturbances in ER homeostasis [30, 31] and thus activation of ER stress and UPR pathway [7]. Few studies have described ER stress responses in MCNs of hypothalamus under physiological conditions [23], but ER stress is believed to be involved in the pathogenesis of familial neurohypophyseal diabetes insipidus (FNDI), a progressive neurodegenerative disease caused by mutations of AVP gene which results in symptoms of polyuria and polydipsia [32, 33]. These mutations interfere with the folding of the AVP precursor protein in MCNs of hypothalamus, resulting in retention of the mutant precursor in the ER lumen [32]. *In vitro* studies in Neuro-2a cells have additionally highlighted that mutations in the AVP gene cause protein aggregation in the ER lumen and alter AVP secretion, with corresponding changes in gene markers for ER stress and apoptosis [33].

The increase in *BiP and Atf4* mRNA expression by hypertonic stress in the SON and PVN, suggests that MCNs were responding to a level of ER stress. An increase in *BiP* mRNA expression in SON and PVN has previously been reported following water deprivation in mice, consistent with our findings here in the rat [23]. Furthermore, a recent study using *Atf6 -/-* knockout mice showed that ER stress gene *Atf6* is important for AVP MCNs in the maintenance of water homeostasis during water deprivation [34]. These evidence support roles of ER stress during hyperosmotic stimulation in the hypothalamus. However no increase of *Xbp1*S, which is a major marker used to detect ER stress activation, in 3 days DH and 7 days SL, suggests that the increased expression of ER stress genes observed in the current study may not result from a fully activated ER stress response. A ‘mild’ level of ER stress activation has previously been described in other physiological events, being aptly named physiological ER stress. Such mild activation of ER stress is believed to help cells to improve their ER function during events that require a high level of protein synthesis and also to facilitate activation of downstream genes that are involved in those physiological events [19–21]. As shown by Murakami and colleagues [20], a mild ER stress is activated during maturation of osteoblasts in bone formation. Unlike classical ER stress, they showed that activation of ER stress in osteoblasts is induced by treatment with bone morphogenetic protein 2. A lower level of ER stress gene activation was observed compared to treatment of classical ER stress inducer thapsigargin. Moreover, they proposed that this physiological ER stress activates CREB3L1 which acts as a transcription factor for synthesis of the Collagen type 1 alpha1 in osteoblasts.

Surprisingly, with no increase of *Xbp1*S, we observed increased *Xbp1*U in SON and PVN in response of hypertonic stress. A recent *in vitro* study in HeLa cells treated with ER stress inducers, tunicamycin and thapsigargin, similarly reported increased *Xbp1*U mRNA expression [35]. They also showed that *Xbp1*U negatively regulated *Xbp1*S by forming a complex with *Xbp1*S, which is then rapidly degraded by proteasomes. Therefore, the increase of *Xbp1*U in SON and PVN observed here may perhaps be a control mechanism to limit the level of ER stress activation in this condition.

In response to ER stress, *Atf4* transcription and translation increase [36]. The phosphorylation of eukaryotic translation initiator factor 2α (eIF2α) by PERK promotes translation of ATF4 which acts as a transcription factor to increase expression of *Chop* [9]. CHOP protein induces reactivation of eIF2α through its target GADD34 (growth arrest and DNA damage-inducible protein) to complete this regulatory loop. In the current study, the consistent increases in *Atf4* mRNA expression in the two hypertonic stimuli, DH and SL, and the parallel changes in *Chop* mRNA expression are consistent with the predicted transcriptional regulation of *Chop* by ATF4. The heterogeneous cell populations in the PVN may explain why we did not see an increase of *Chop* in this nucleus. CHOP is also known as a pro-apoptotic protein that links the ER stress pathway to apoptosis. It functions in down-regulation of the anti-apoptotic protein BCL-2 and up-regulation of GADD34 which induces the generation of reactive oxygen species, leading to apoptosis [8]. However, no evidence of apoptosis has been reported in AVP MCNs in response to hyperosmotic stress, although AVP neuronal loss has been reported in late stage-FNDI mice [32].

A relatively new component to the ER stress network is transcription factor CREB3L1. Expression and activation of CREB3L1 by ER stress has been reported in a number of different organs and systems, including central nervous system [20, 37, 38]. We recently showed that CREB3L1 regulates AVP transcription in the hypothalamus [22], but the relationship between CREB3L1 and ER stress in MCNs of the SON and PVN has not been investigated before. To investigate possible roles of CREB3L1 in ER stress we used a lentivirus system to over-express CREB3L1DN in the SON of rats subjected to 3 days DH in order to knockdown endogenous CREB3L1 activity [37]. CREB3L1 has previously been reported to modulate the UPR response in astrocytes by inducing transcription of ER stress gene, *Bi*P [37]. Here we show that over-expression CREB3L1DN in the dehydrated SON had no effect on *BiP* mRNA expression. In support of our findings a previous study in bone tissue and osteoblasts of *Creb3l1*-knockout mice also reported no change in the expression of ER stress-related genes including *BiP* [20]. This agrees with cell type-specific functions of CREB3L1, as previously reported [38, 39].

Using CREB3L1DN lentiviral vector injected into the SON, we showed that CREB3L1 could regulate the expression of ER stress genes *Chop* and *Xbp1*U, but the expression of *BiP* and *Atf4*, two important marker of ER stress, were not altered. The expression of *Xbp1* is directly controlled by ATF6 [11], a transcription factor with similar structure to CREB3L1, implying that CREB3L1 may similarly regulate *Xbp1* transcription [39, 40]. Furthermore, there is emerging evidence for novel outputs of the UPR in many physiological processes beyond the homeostasis of protein folding [8]. For example, Toll-like receptor signalling was shown to specifically activate *Xbp1*. In the absence of activation of the other branches of ER stress pathway, *Xbp1* functions to enhance production of cytokines such as interleukin-6, not ER stress response [41]. Therefore, regulation of *Chop* and *Xbp1*U by CREB3L1 may have a role in the novel UPR output, not a classical ER stress response. Immunofluorescent staining showed co-existence of CHOP with AVP and CREB3L1, supporting a functional relationship between these proteins in AVP MCNs, although further investigations is necessary to unravel the exact molecular mechanisms.

In summary, this study provides evidence for the activation of ER stress genes in the SON and PVN in response to physiological stimuli such as water deprivation and intake of hypertonic saline solution in the rat. This supports a role for physiological ER stress in these brain nuclei to meet the large demands for protein synthesis during hypertonic stress. By over-expressing CREB3L1DN in the SON we have also provided evidence that CREB3L1 may function to regulate components of the ER stress pathways in the MCNs of the osmotically stimulated hypothalamus.
